# Supplementary material for: LCK facilitates DNA damage repair by stabilizing RAD51 and BRCA1 in the nucleus of chemoresistant ovarian cancer
Source: J Ovarian Res. 2023 Jun 27;16:122. doi: 10.1186/s13048-023-01194-2 (PMC10294509; doi:10.1186/s13048-023-01194-2)
Supplement: Supplementary file 1 — Additional file 1: Supplementary Fig. S1. Transfection efficiency of Myc tagged LCK in SKOV3 and CP70. SKOV3 and CP70 cells were transduced with EV or Myc tagged LCK plasmid by using lentiviral particle. Then, cells were checked for Myc and LCK expression. Supplementary Fig. S2. CP70 EV and CP70 LCK OE cells were treated with cycloheximide in a time dependent manner. Then, immunoblot analysis was performed to evaluate the expression of RAD51 and BRCA1 proteins (Main Fig. 1G, H). Half-lives were determined from digitized images. Supplementary Fig. S3. (A) CP70 WT, LCK KO (CRISPR/Cas9) and LCK OE (In CRISPR background) cells were treated with DMSO/etoposide 10µM for 24h. Then cells were kept in drug free media for another 24h. Then immunofluorescence study was performed to visualize γH2AX foci formation in different groups. (B) CP70 WT, LCK KO (CRISPR/Cas9) and LCK OE (In CRISPR background) cells were treated with DMSO/etoposide 10µM for 24h. Cells were put in drug free media for another 24h. Then immunofluorescence study was performed to visualize RAD51 foci formation in different groups. Supplementary Fig. S4. (A) CP70 cells (LCK OE, LCK Y394F, LCK K273R, and LCK Y192F, all constructs were introduced into the CP70 LCK KO cells) were treated with etoposide for 24h. Cells were then kept in drug free media for 24h. Immunofluorescence study was performed to visualize γH2AX foci formation. (B) CP70 cells (LCK OE, LCK Y394F, LCK K273R, and LCK Y192F) were treated with etoposide for 24h. Cells were then kept in drug free media for 24h. Immunofluorescence study was performed to visualize RAD51 foci formation. Supplementary Fig. S5. CP70 cells (LCK, LCK Y394F, LCK K273R, and LCK Y192F in LCK knock out background) were grown on cover slips and treated with etoposide for 24h followed by incubation for 0, 2, 4, 8 and 24h. Cells were then subjected to immunofluorescence analysis to visualize H2AX foci formation. Supplementary Fig. S6. (A) SKOV3 cells were treated with etoposide, [file 13048_2023_1194_MOESM1_ESM.docx]

***Supplementary File***

***LCK Facilitates DNA Damage Repair by Stabilizing RAD51 and BRCA1 in the Nucleus of Chemoresistant Ovarian Cancer***

Goutam Dey^1+^, Rashmi Bharti^1+^, Chad Braley^1^, Ravi Alluri^1^, Emily Esakov^1^, Katie Crean-Tate^2^, Keith McCrae^1,5^, Amy Joehlin-Price^3^, Peter G. Rose^2^, Justin Lathia^1,5^, Zihua Gong^4,5^, Ofer Reizes^1,5*^

**Supplementary Fig. S1**


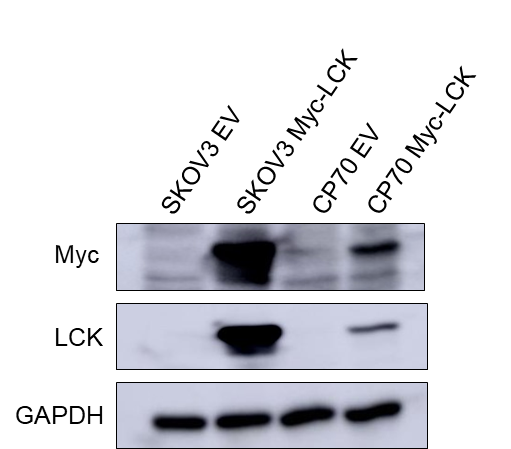


**Supplementary Fig. S1:** Transfection efficiency of Myc tagged LCK in SKOV3 and CP70. SKOV3 and CP70 cells were transduced with EV or Myc tagged LCK plasmid by using lentiviral particle. Then, cells were checked for Myc and LCK expression.


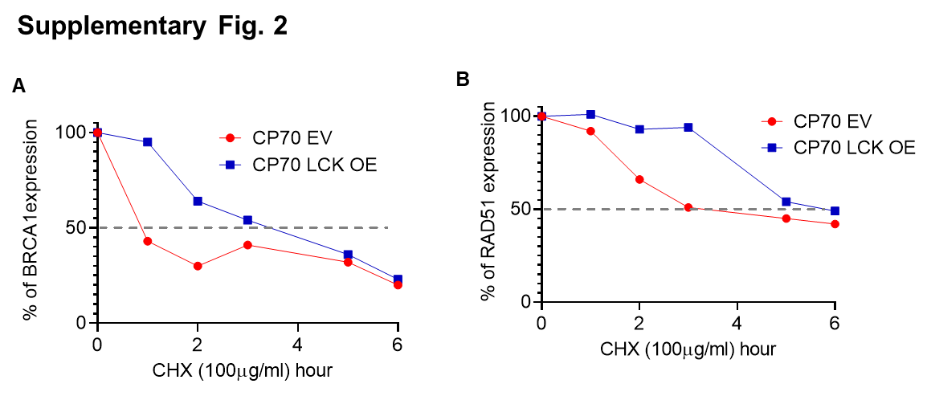


**Supplementary Fig. S2:** CP70 EV and CP70 LCK OE cells were treated with cycloheximide in a time dependent manner. Then, immunoblot analysis was performed to evaluate the expression of RAD51 and BRCA1 proteins (Main Fig. 1G, H). Half-lives were determined from digitized images.

**
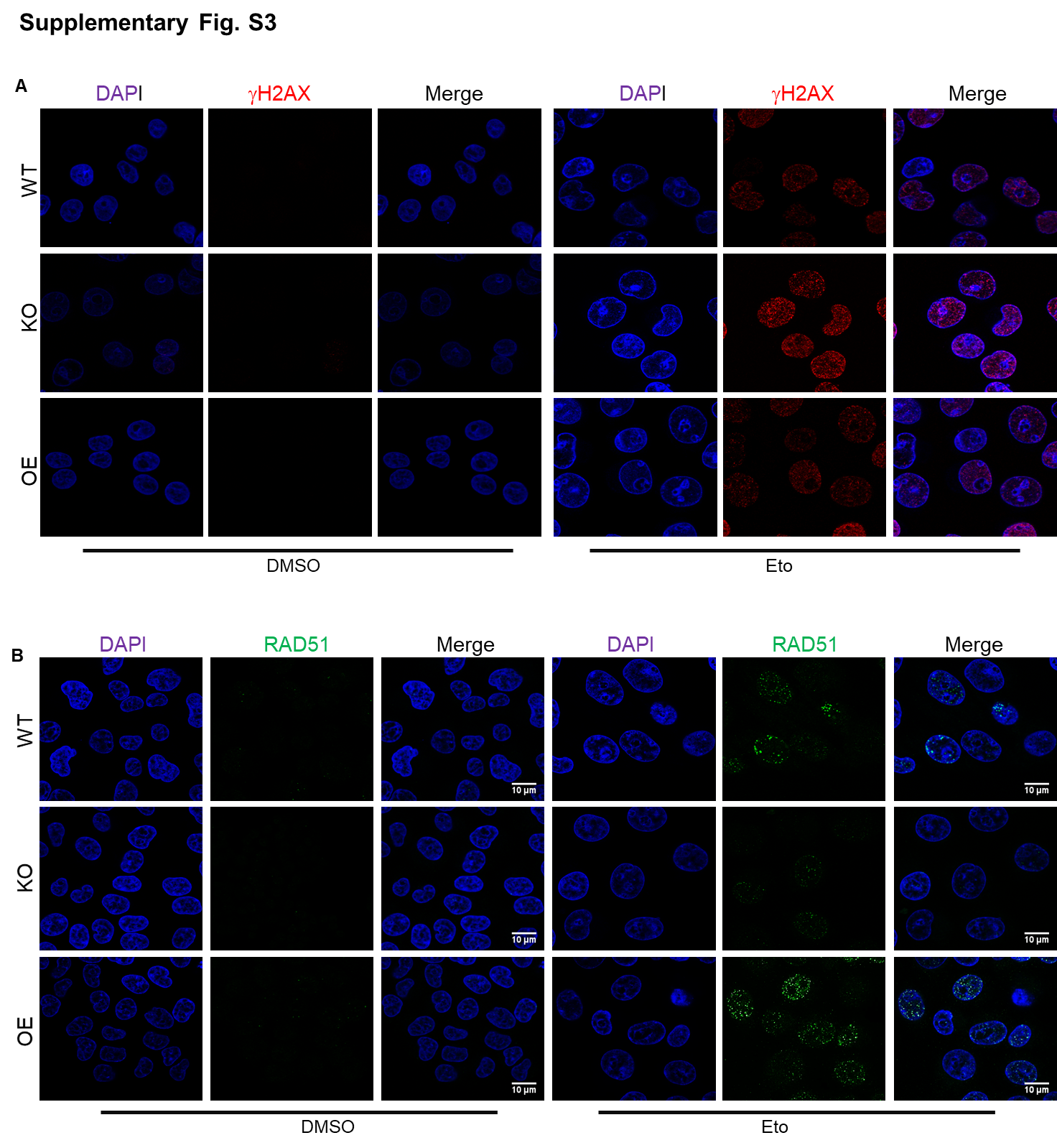
Supplementary Fig. S3 (A)** CP70 WT, LCK KO (CRISPR/Cas9) and LCK OE (In CRISPR background) cells were treated with DMSO/etoposide 10µM for 24h. Then cells were kept in drug free media for another 24h. Then immunofluorescence study was performed to visualize γH2AX foci formation in different groups. **(B)** CP70 WT, LCK KO (CRISPR/Cas9) and LCK OE (In CRISPR background) cells were treated with DMSO/etoposide 10µM for 24h. Cells were put in drug free media for another 24h. Then immunofluorescence study was performed to visualize RAD51 foci formation in different groups.

**
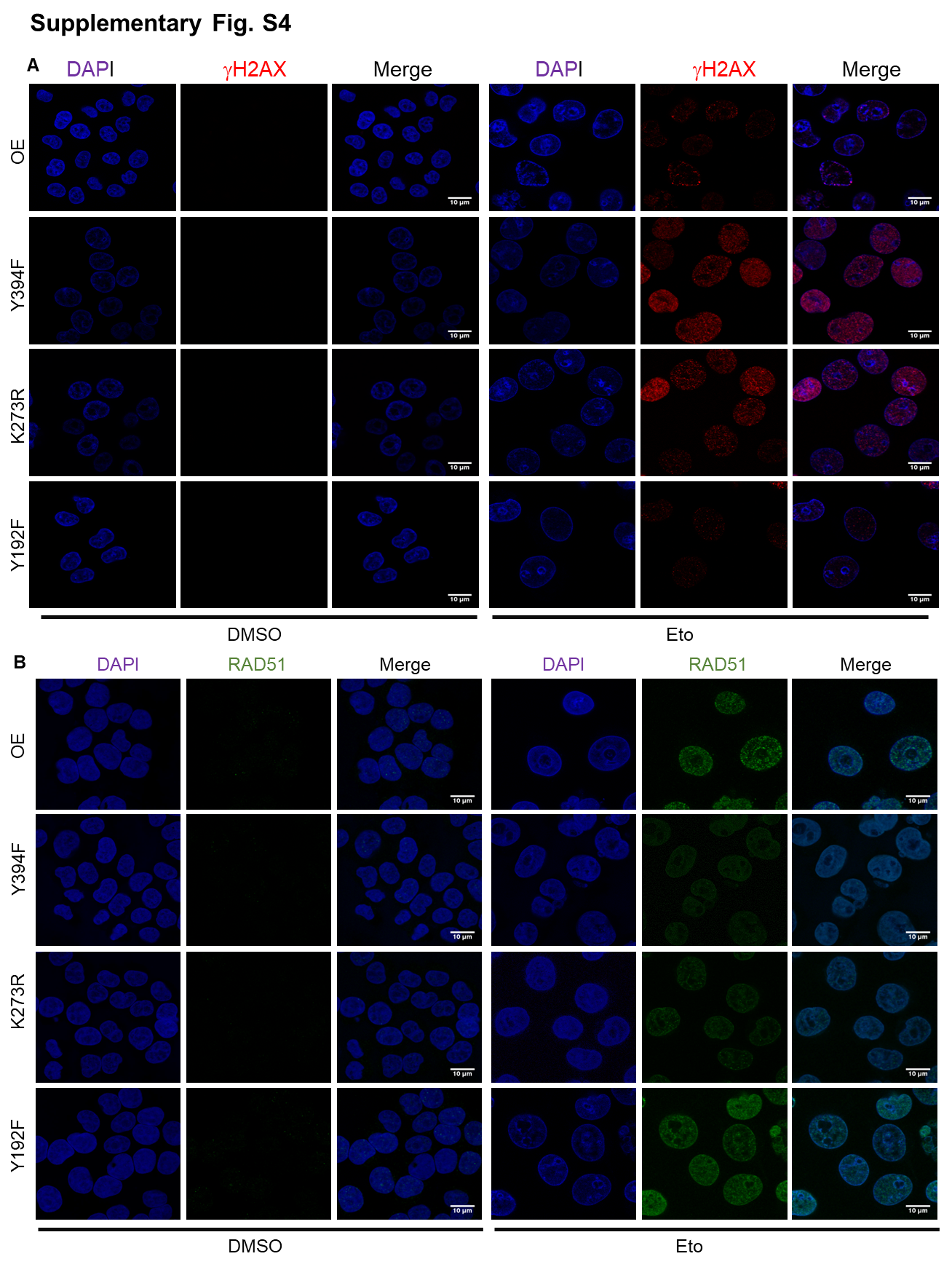
Supplementary Fig. S4: (A)** CP70 cells (LCK OE, LCK Y394F, LCK K273R, and LCK Y192F, all constructs were introduced into the CP70 LCK KO cells) were treated with etoposide for 24h**.** Cells were then kept in drug free media for 24h. Immunofluorescence study was performed to visualize γH2AX foci formation. **(B)** CP70 cells (LCK OE, LCK Y394F, LCK K273R, and LCK Y192F) were treated with etoposide for 24h**.** Cells were then kept in drug free media for 24h. Immunofluorescence study was performed to visualize RAD51 foci formation.

**
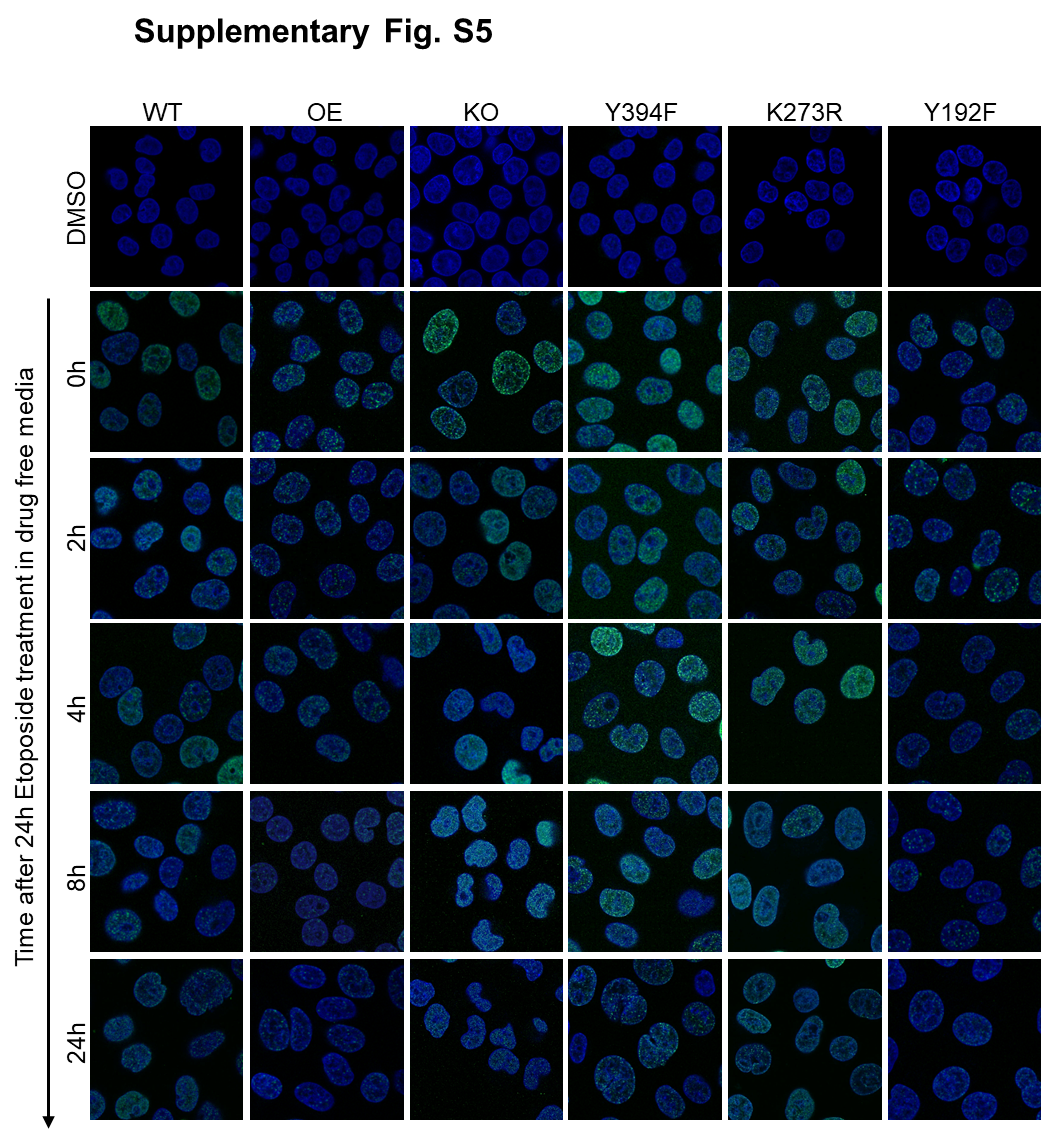
**

**Supplementary Fig. S5:** CP70 cells (LCK, LCK Y394F, LCK K273R, and LCK Y192F in LCK knock out background) were grown on cover slips and treated with etoposide for 24h followed by incubation for 0, 2, 4, 8 and 24h. Cells were then subjected to immunofluorescence analysis to visualize H2AX foci formation.

**
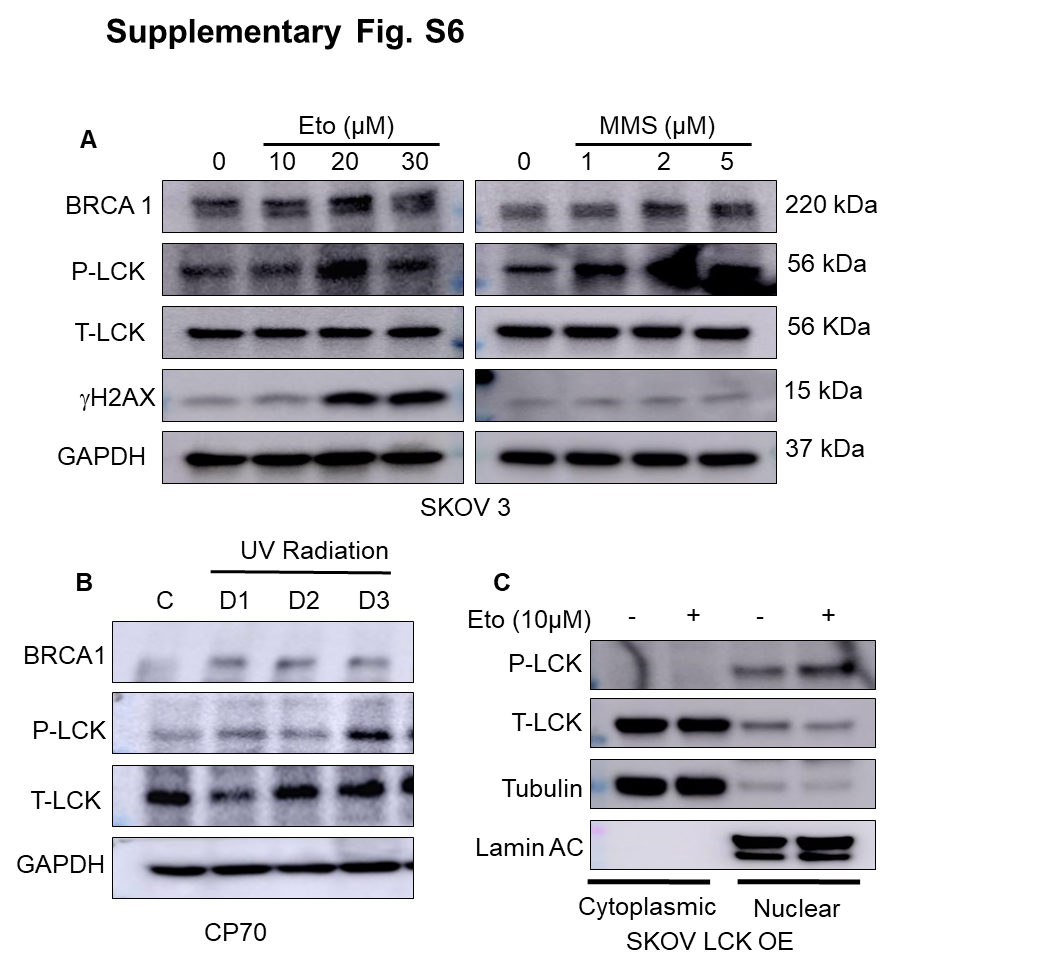
**

**Supplementary Fig. S6**: **(A)** SKOV3 cells were treated with etoposide, and MMS for 24. After that cells were put in 24h in drug free media. Cells were then subjected to western blot analysis for checking protein expression. **(B)** DNA damage by UV radiation upregulates LCK phosphorylation. CP70 cells were treated with UV radiation for 1min, 2min and 4min. Cells were kept in serum enriched media for 24h. Then cells were subjected to western blot analysis to check the expression of P-LCK, T-LCK and BRCA1 expression. **(C)** SKOV3 LCK OE cells were treated with etoposide for 24h. Cells were then put in drug free media for another 24h. Cells were collected, and cytoplasmic and nuclear proteins were extracted for western blot analysis.

**
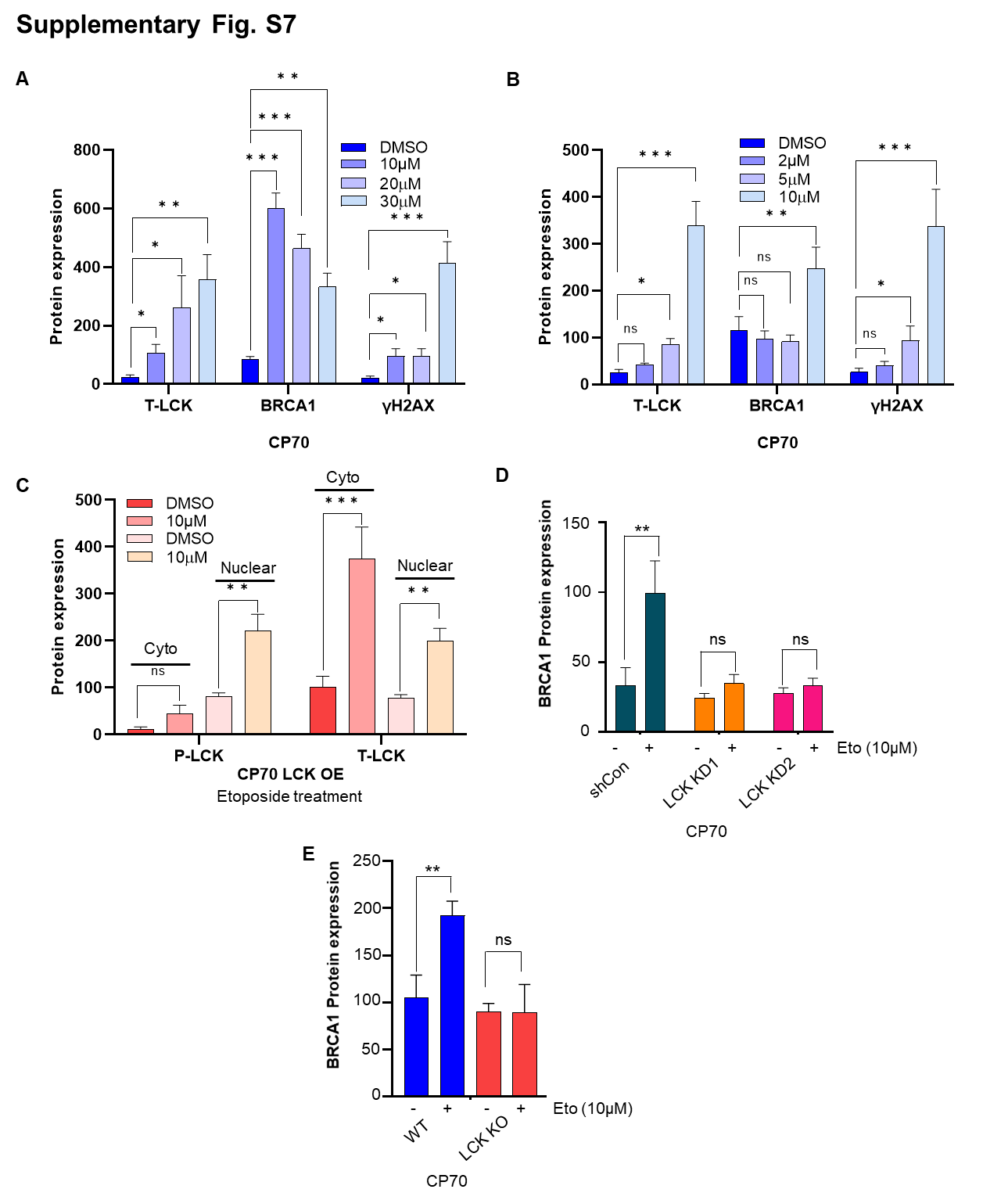
**

**Supplementary Fig. S7 (A, B)** Protein quantification of T-LCK, BRCA1, and γH2AX in CP70 cells treated with etoposide and MMS (Main Fig. 2A). **(C)** Quantification of P-LCK and T-LCK in CP70 LCK OE cells treated with etoposide (Main Fig. 2B). **(D)** Quantification of BRCA1 protein in CP70 shCon, LCK KD1 and LCK KD2 cells treated with/without etoposide (Main Fig. 3D). **(E)** Quantification of BRCA1 protein expression in CP70 WT and LCK KO cells treated with/without etoposide (Main Fig. 3E). One way ANOVA analysis was performed with Tukey's multiple comparisons test to compare different groups (* p < 0.05, ** p < 0.01, *** p < 0.001).

**
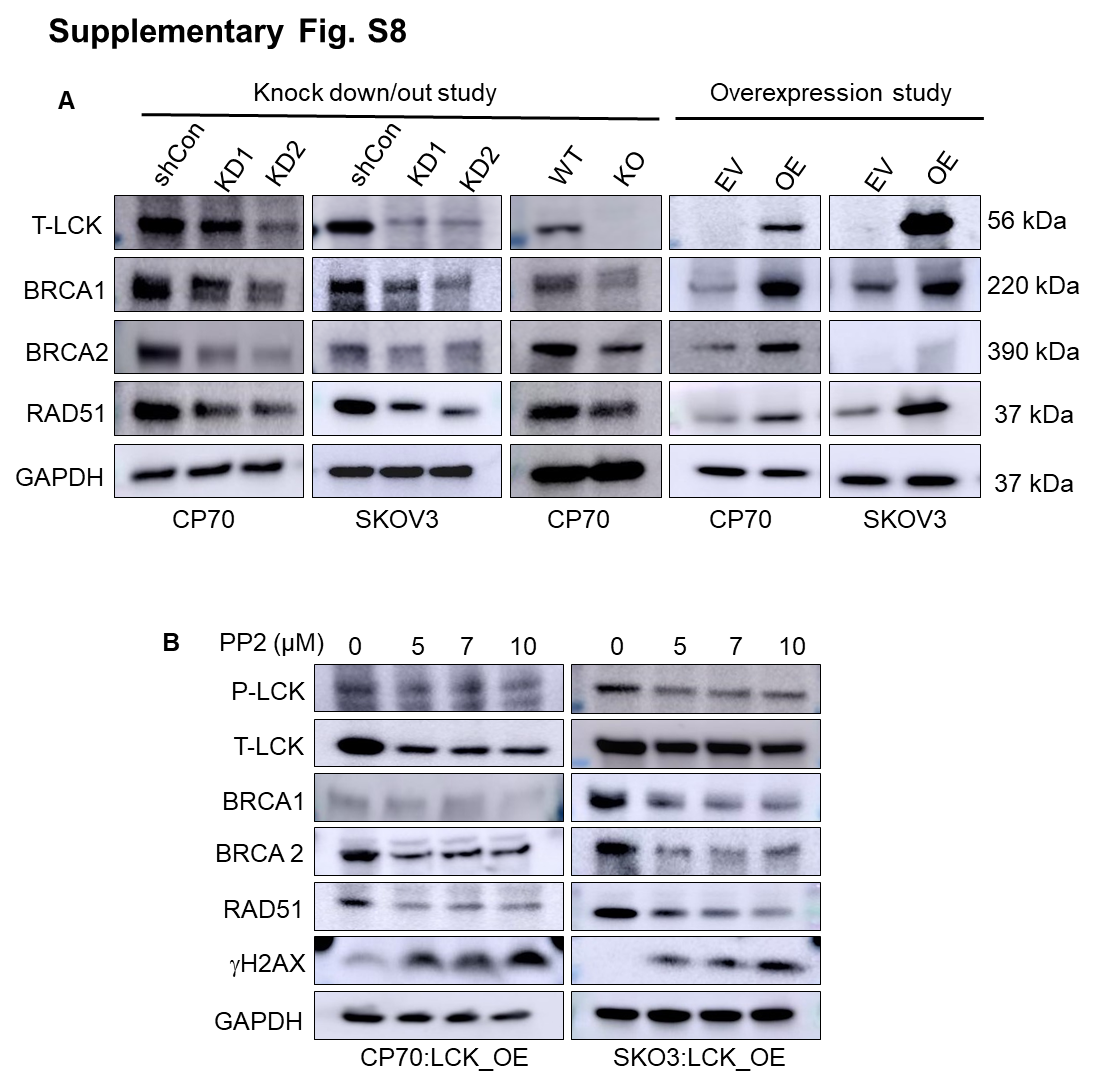
**

**Supplementary Fig S8: LCK modulates expression of HR repair proteins. (A)** Western blot of CP70 and SKOV3 cells containing various lentiviral EV, LCK KD, KO, and OE to determine effects on LCK, BRCA1, BRCA2 and RAD51 expression. We detected a significant basal level of LCK in WT cells. In EV-OE panel, we used empty vector and LCK OE plasmid transduced in LCK knock out cells. **(B)** Western blot analysis of CP70 and SKOV3 LCK OE cells treated with PP2 in a dose dependent manner for 48hrs, demonstrating the effects of a pharmacological inhibitor of LCK on P-LCK, T-LCK, BRCA1, BRCA2, RAD51 and γH2AX protein expression.


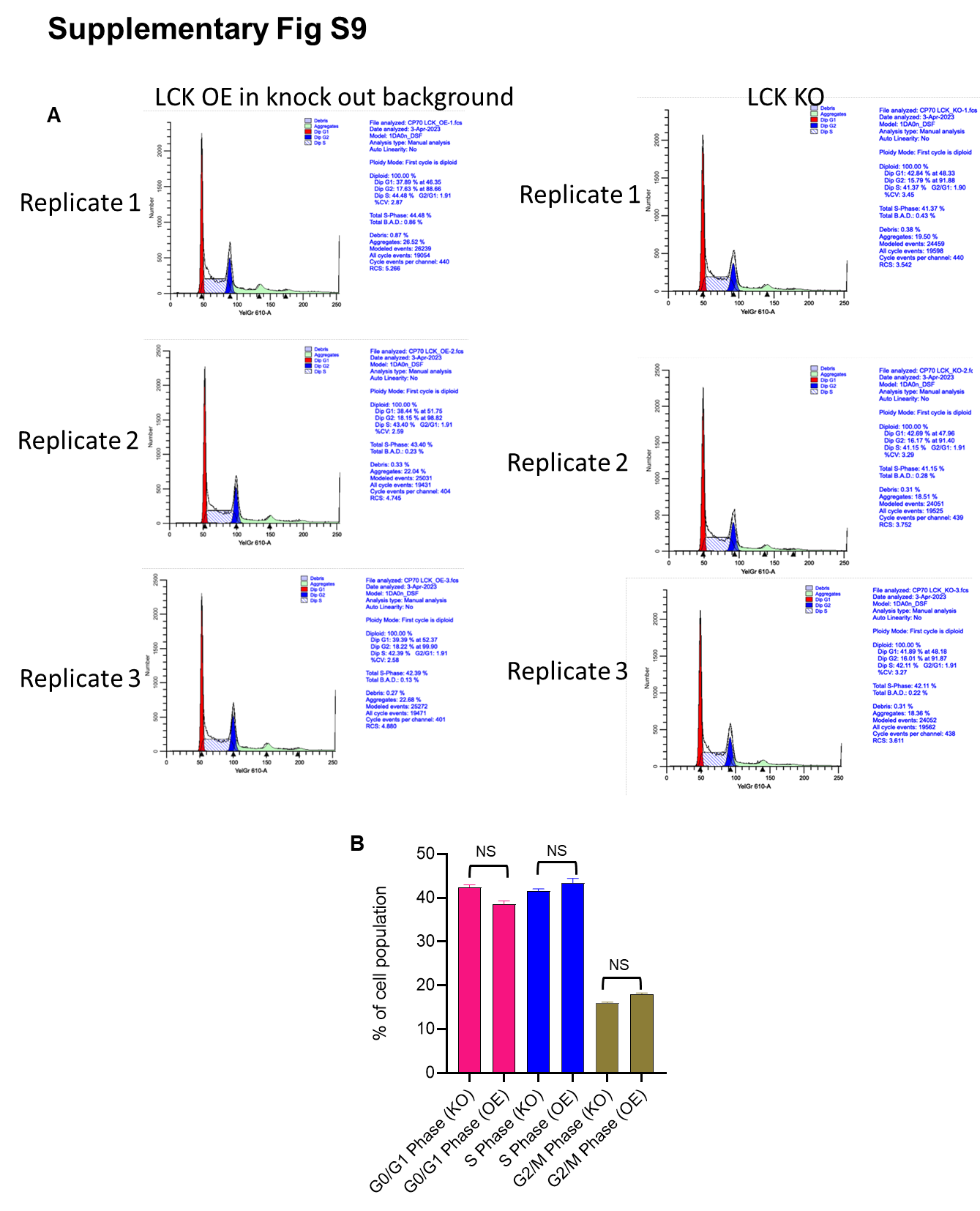


**Supplementary Fig S9**: Cell cycle analysis by flow cytometry. (A) CP70 cells (LCK OE and KO) were grown to 70% confluency. Cells were harvested by trypsinization and fixed with chilled ethanol. Cell were then stained with propidium iodide and subjected to flow cytometry. Experiments were replicated three times. (B) Percentage of cell population in each phase of cell cycle are presented. One way ANOVA analysis was performed with Tukey's multiple comparisons test to compare different groups (* p < 0.05, ** p < 0.01, *** p < 0.001).

**
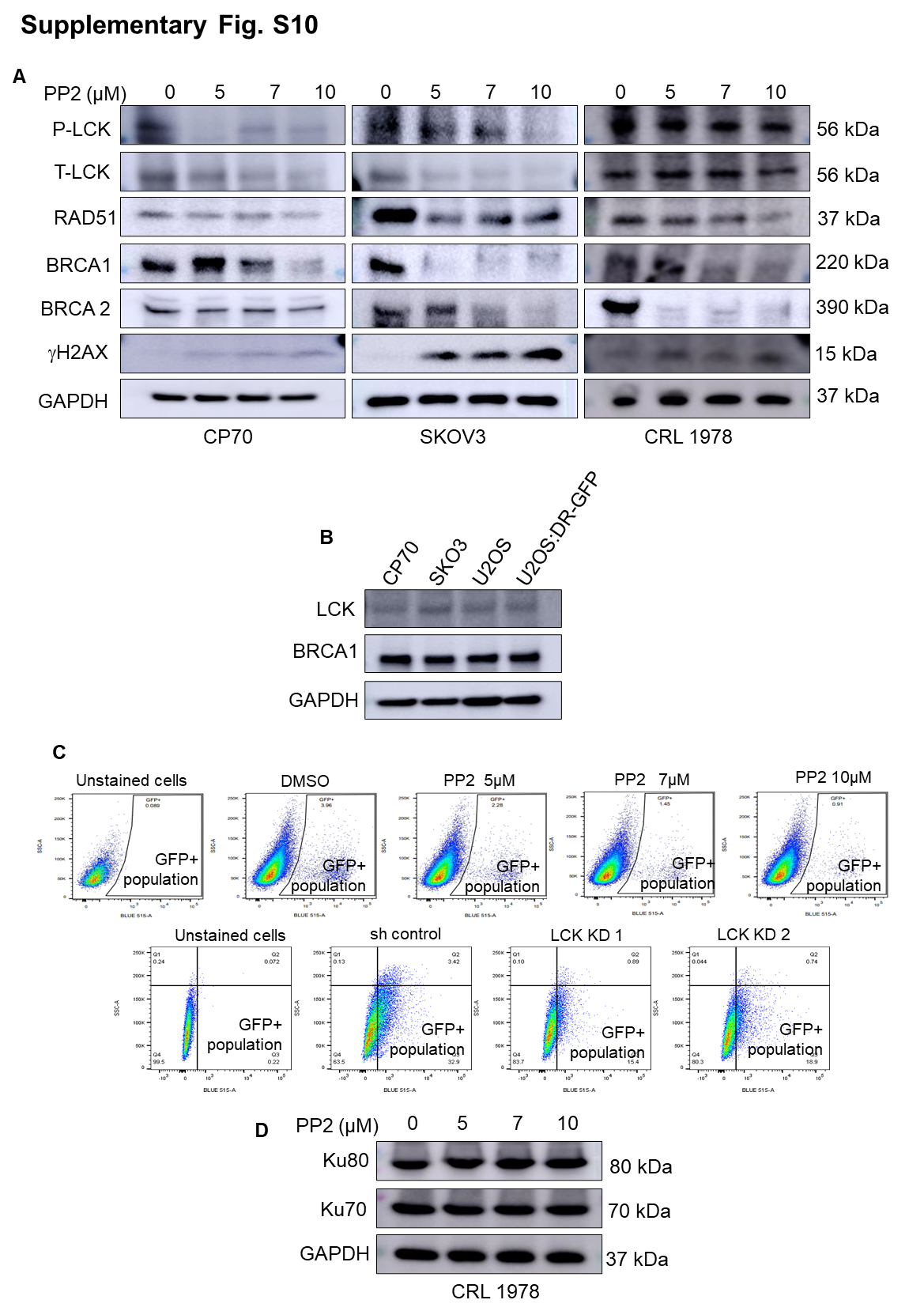
**

**Supplementary Fig. S10: (A)** Pharmacological inhibition of LCK attenuates HR repair proteins in ovarian cancer cells. CP70, SKOV3 and CRL1978 cells were treated with the LCKi, PP2 for 48h. Cells were harvested, lysed, and analyzed by immunoblot to assess protein expression of BRCA1, BRCA2, RAD51, and γH2AX. **(B)** Western blot analysis in different cells to check the expression of LCK and BRCA1. U2OS is osteosarcoma cell line which was used in DR GFP assay. U2OS and U2OS: DR-GFP cells were examined for checking LCK and BRCA1 expression. These cells were also found to express LCK and BRCA1 like CP70 and SKOV3 cells. **(C)** Representative image of flow cytometry from DRGFP assay. Upper panel is shows GFP population of U2OS cells treated with PP2 and lower panel is shows GFP population in Sh Control, LCK KD1 and KD2 groups of U2OS cells. **(D)** CRL1978 cells were treated with increasing concentrations of PP2 for 48h and cells were harvested, lysed, and immunoblotted for Ku70, and Ku80 protein expression. GAPDH was used as loading control.


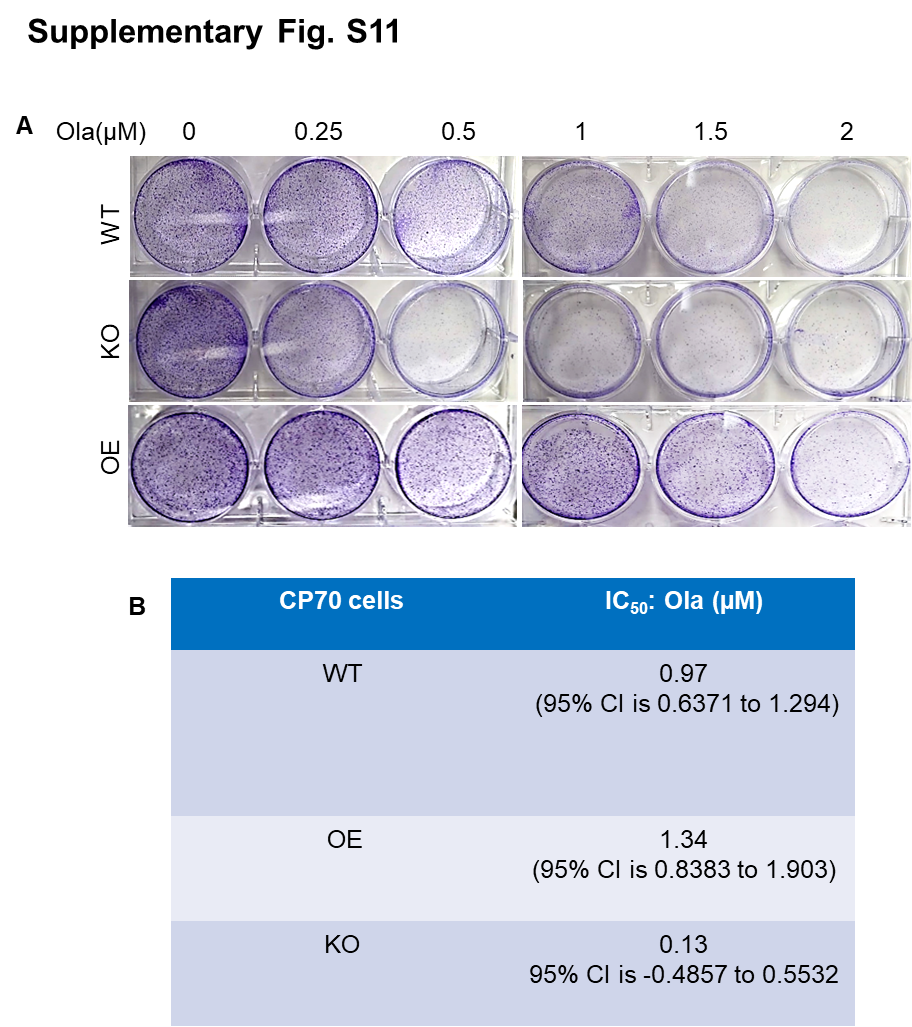


**Supplementary Fig. S11: (A)** CP70 Parental cells and CP70 LCK KO and CP70 CD55 OE (In KO background) cells were treated with Olaparib in dose dependent manner for 12 days. After that colonies were stained with crystal violet and images were captured. **(B)** Number of Colony formation was counted and plotted as percentage of colony formation in the graph (Main fig 6G). IC50 values were shown in the table.

**
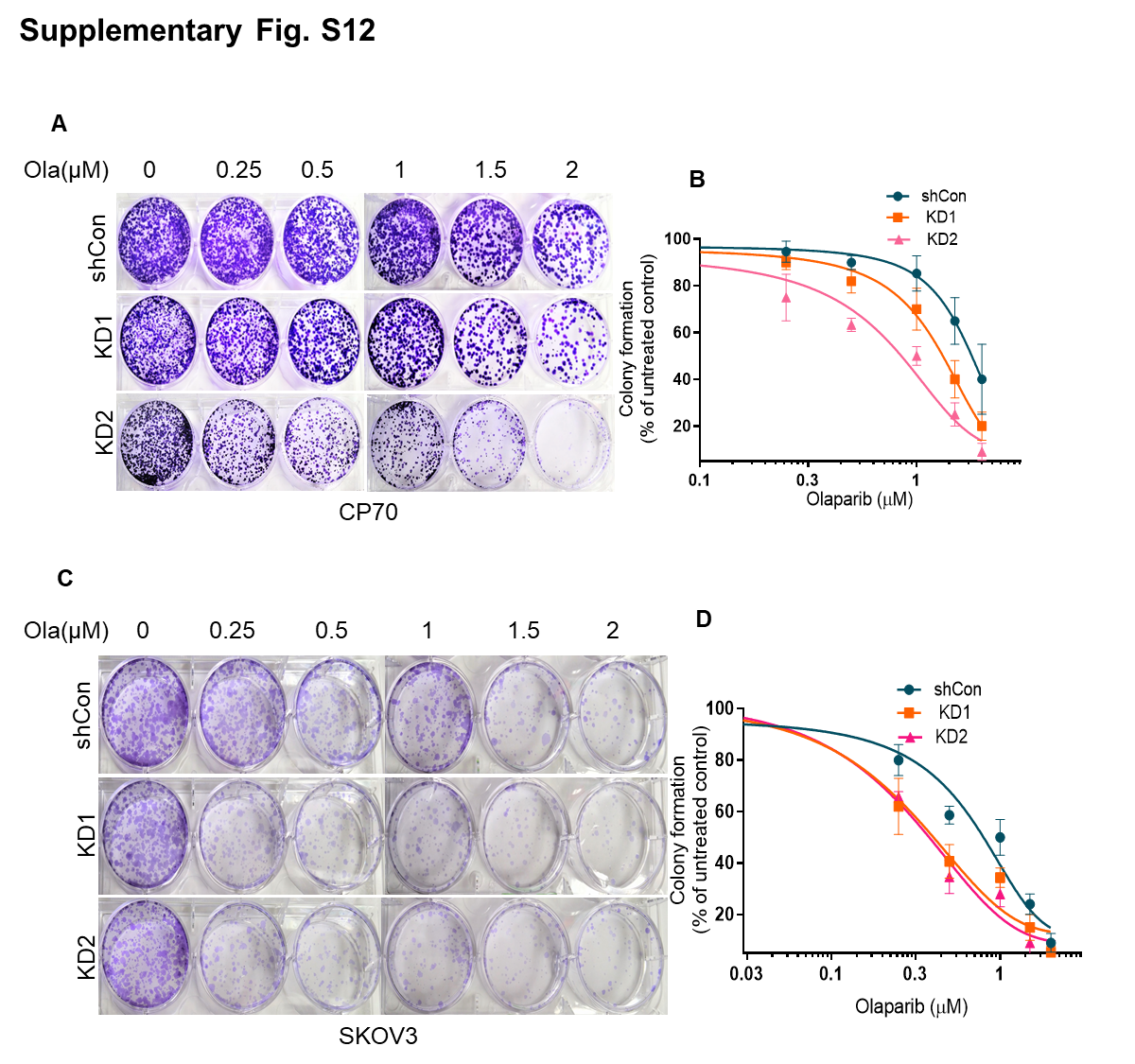
**

**Supplementary Fig. S12: (A, B)** CP70 Sh Con or LCK knock down cells were treated with Olaparib in a dose dependent manner for 12 days. Number of colonies was counted and plotted using graph pad prism. **(C, D)** SKOV3 Sh Con or LCK knock down cells were treated with Olaparib in dose dependent manner for 12 days. Number of Colony formation was counted and plotted in the graph.

**
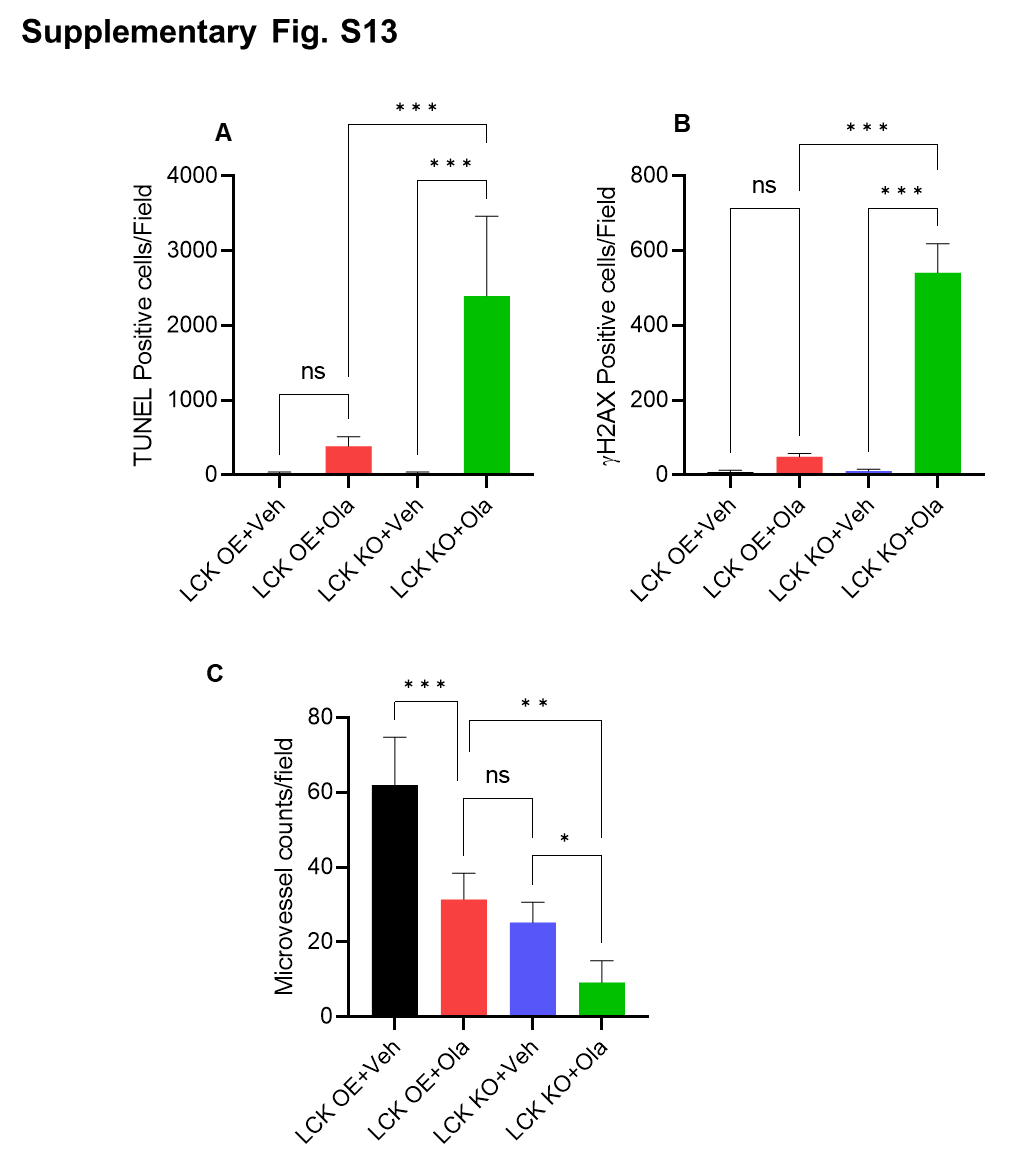
**

**Supplementary Fig. S13: (A)** TUNEL assay to detect DNA fragmentation in tumor tissue sections. TUNEL positive cells were counted from five images and plotted in graph (Main fig. 8D). **(B)** IHC staining of γH2AX of tumor sections from different groups. γH2AX positive cells were counted from five images and plotted in graph (Main fig. 8E).). **(C)** CD31 expression (Indicator of microvessel density and growth) of tumor sections from different group of mice. Microvessel density was counted from five images and plotted in graph (Main fig. 8F). Images are representative of two tumors from each cohort. We quantified the staining from 5 fields from each mouse. Images were captured at 20X magnification. One way ANOVA analysis was performed with Tukey's multiple comparisons test to compare different groups (* p < 0.05, ** p < 0.01, *** p < 0.001).

**Supplementary table S14**

| REAGENT or RESOURCE | SOURCE | IDENTIFIER |
| --- | --- | --- |
| Antibodies |  |  |
| Anti-LCK antibody | Proteintech | 12477-1-AP |
| Anti-LCK antibody | R&D System | MAB3704 |
| Anti-P-LCK (Y394) antibody | R & D system | MAB7500 |
| Anti-RAD51 antibody | Proteintech | 14961-1-AP |
| Anti-RAD51 antibody | Santa Cruz | sc-398587 |
| Anti-P-H2A.X (Ser139) Antibody | Cell signaling | 2577S |
| Anti-BRCA1 antibody | EMD Millipore | OP-92-100UG |
| Anti-BRCA2 antibody | EMD Millipore | OP-95-100UG |
| Anti-Myc antibody | Proteintech | 60003-2-Ig |
| Anti-GAPDH antibody | Proteintech | HRP-60004 |
| Anti-Lamin A/C antibody | Proteintech | 10298-1-AP |
| Alpha Tubulin Monoclonal antibody | Proteintech | HRP-66031 |
| Anti-Ku70 antibody | Proteintech | 10723-1-AP |
| Anti-Ku80 antibody | Proteintech | 16389-1-AP |
| Rabbit IgG XP® Isotype Control | Cell signaling | 3900S |
| Mouse IgG XP® Isotype Control | Cell signaling | 5415S |
| Anti-Rabbit IgG (H+L), HRP antibody | Promega | W4018 |
| Anti-Mouse IgG (H+L), HRP antibody | Promega | W4028 |
| Goat anti-Rabbit IgG Alexa Fluor 488 | Thermo | A32731 |
| Goat anti-mouse IgG Alexa Fluor 568 | Thermo | A11031 |
| Chemicals and reagents |  |  |
| Precision Plus Protein™ Kaleidoscope | Biorad | 1610375 |
| Olaparib | Selleck chemicals | S1060 |
| PP2 | Selleck chemicals | S7008 |
| Etoposide | Selleck chemicals | S1225 |
| Cisplatin | Fesenius Kabi | 401572I |
| Colcemid | Sigma | 10295892001 |
| Laemmli SDS sample buffer, reducing (6X) | Alfa Aesar | J61337 |
| Pierce™ Protein A/G Plus Agarose | Thermo | 20423 |
| Precision Plus Protein™ Kaleidoscope™ | 1610375 | Biorad |
| SYBR™ Gold Nucleic Acid Gel Stain | Thermo | S11494 |
| Trypan Blue | Fisher scientific | 25900CI |
| Immobilon-P PVDF Membrane | Merck Millipore | IPVH00010 |
| Critical Commercial Assays |  |  |
| VECTASHIELD^®^ Mounting Medium | Vector lab | H-1200 |
| NP40 Lysis buffer | Thermo | FNN0021 |
| Pierce™ IP Lysis Buffer | Thermo | 87788 |
| Comet Assay Kit | Trevigen | 4250-050-K |
| NuPAGE™ Protein Gel | Thermo | NP0329BOX |
| 4–20% Mini-PROTEAN Protein Gels | Biorad | 4568096 |
| Pierce BCA Protein Assay Kit | Thermo | 23225 |
| Lipofectamin 3000 | Thermo | L3000001 |
| CellTiter-Glo® 2.0 Cell Viability Assay | Promega | G9241 |
| Live/Dead assay kit | Thermo | L23105 |
| KaryoMAX™ Giemsa Stain Solution | Thermo | 10092013 |
| Giemsa Stain, Modified Solution | 32884-1L | Sigma |
| NP40 lysis buffer | Invitrogen | FNN0021 |
| Protease Inhibitor Cocktail | Sigma | 04693159001 |
| Cytoplasmic and nuclear protein isolation kit | Thermo | 78835 |
| Experimental Models: Cell Lines |  |  |
| CP70 | Dr. Analisa Difeo | NA |
| SKOV3 | ATCC | NA |
| U2OS | Dr. Zihua Gong | NA |
| U2OS DRGFP reporter cells | Dr. Zihua Gong | NA |
| HEK293T | ATCC | NA |
| CRL1978 | ATCC | NA |
| Recombinant DNA |  |  |
| pLenti CMV Puro DEST | Addgene | NA |
| Myc-LCK pLenti CMV Puro DEST | In house |  |
| Myc-LCK Y394F pLenti CMV Puro DEST | In house | NA |
| Myc-LCK K273R pLenti CMV Puro DEST | In house | NA |
| Myc-LCK Y192F pLenti CMV Puro DEST | In house | NA |
| I-SceI plasmid | Dr. Zihua Gong | NA |
| ShRNA targeting LCK | Sigma Aldrich | TRCN0000426292 |
| ShRNA targeting LCK | Sigma Aldrich | TRCN0000001600 |
| ShRNA targeting LCK | Sigma Aldrich | TRCN0000001598 |
| ShRNA targeting LCK | Sigma Aldrich | TRCN0000001599 |
| LCK CRISPR/Cas9 KO Plasmid | Santa Cruz | SC-400434-KO-2 |
| Software |  |  |
| FlowJo | BD Bioscience | NA |
| Graph Pad prism | www.graphpad.com | NA |
| ImageJ | imagej.nih.gov | NA |

**Supplementary table S15**

| Cells | IC_50_ of Etoposide (μM) | 95% Confidence Interval (CI) |
| --- | --- | --- |
| CP70 | 5μM | 4.268 to 5.682 |
| CP70 LCK KO | 1.67μM | 1.48 to 1.90 |
| CP70 LCK OE | 10.03μM | 7.894 to 12.67 |
| CP70 LCK Y192F | 8.70μM | 7.044 to 10.72 |
| CP70 LCK K273R | 2.28μM | 1.938 to 2.692 |
| CP70 LCK Y394F | 2.01μM | 1.655 to 2.455 |
